# Characterization of patients admitted to intensive care unit directly from emergency department

**DOI:** 10.1186/1757-7241-20-S2-P8

**Published:** 2012-04-16

**Authors:** Anja Lindén, Willy Krone, Thomas A Schmidt

**Affiliations:** 1 Emergency Department, Holbæk sygehus, Denmark

## Background

Some patients admitted to the Emergency Department (ED) are so ill, that they are referred directly to the intensive care unit (ICU).

We wished to estimate which medical conditions require direct admission from the ED to the ICU, their treatment, and the average length of stay.

## Methods

We looked at patients admitted from the ED to the ICU within a period of three months (Feb 1st – April 30th 2011).

We included paediatric patients in need of surgical treatment and all adults, except for those suffering a trauma or with gynaecological and obstetric conditions.

## Results

In the course of three months, 49 patients were admitted to the ICU via the ED.

Average length of admission was 3.1 days. Main conditions were divided into seven subgroups; drug intoxication (n=13), respiratory insufficiency (n=11), GI-haemorrhage (n=6), high-pressure lung oedema (n=3), electrolyte disturbances (n=3), septic shock (n=3) and others (n=10).

Treatments primarily used were, fluid/electrolyte therapy (n=29), broad-spectrum antibiotics (n=24), intubation (n=12), blood component therapy (n=11), NIV treatment (n=10), corticosteroids (n=10), beta-agonist inhalation treatment (n=7), vasopressor drugs (n=8), activated charcoal (n=9), diuretics (n=10), and others (n=52).

In the subgroup of drug intoxication, 69% patients received activated charcoal. The remaining 31% were either unconscious and could not cooperate to place a gastric tube/drink the charcoal, or came to the ED too late, with activated charcoal having no effect. 69% of the patients also expressed that the incident was an attempt at suicide.

## Conclusion

Drug intoxication and respiratory insufficiency accorded for nearly half of all submitted patients to the ICU.

Main treatments given were fluid/electrolyte therapy, 59%, and broad-spectrum antibiotics, 49%. Patients that required management of airways and ventilation added up to 59%, (intubation, NIV and beta-agonist treatment).

Many of the patients’ receiving fluid/electrolyte therapy, and broad-spectrum antibiotics had respiratory insufficiency as the primary cause of admission to the ICU, with infection or suspicion of infection as the underlying cause. This explains why septic shock only accounts for three patients.

In everyday work in an ED, these results can be used as a guide to recognize which groups of patients may need intensive care treatment.

